# Surgical Management of Kartagener's Syndrome With Bronchiectasis in a Pediatric Patient: A Case Report on Right Lower Lung Lobectomy in a 9‐Year‐Old Female

**DOI:** 10.1002/ccr3.71963

**Published:** 2026-02-03

**Authors:** Pakeezah Tabasum, Makhdooom Bilawal, Priya Devi, Waseem Sajjad, Ali Raza Brohi, Mohammed Hammad Jaber Amin

**Affiliations:** ^1^ Peoples University of Medical and Health Sciences for Women Nawabshah Pakistan; ^2^ Ziauddin University Karachi Pakistan; ^3^ Liaqat University of Medical and Health Sciences Jamshoro Pakistan; ^4^ King Edward Medical University Lahore Pakistan; ^5^ Institute of Mother and Child Health Nawabshah Pakistan; ^6^ Alzaiem Alazhari University Khartoum Sudan

**Keywords:** bronchiectasis, Kartagener's syndrome, primary ciliary dyskinesia, situs inversus, thoracoscopic lobectomy

## Abstract

Kartagner syndrome is a rare congenital autosomal recessive disorder of ciliary movement, characterized by triad of chronic sinusitis, situs inversus and bronchiectasis leading to recurrent chest and sinuses infections. The primary objectives of this case report is to highlight the presentation of this rare disorder, its surgical challenge with minimally invasive procedures, and contribute to literature of this rare syndrome in pediatric patient of 9‐year‐old female presented with a complaint of high grade fever and productive cough for 3 days. The X‐ray findings showed Dextrocardia and right sided stomach gas, suggesting Situs inversus. The chest computerized tomography revealed right side apex beat, aortic arch on right side and right lower lobe bronchiectasis. Considering these clinical signs and radiological examination, the patient was diagnosed with Kartagener's syndrome. During Hospitalization, along with conservative treatment, the patient had undergone successful thoracoscopic right lower lobectomy for bronchiectasis secondary to Kartagener's syndrome. This case report presents a rare disorder, highlights the importance of considering Kartagener's syndrome as a differential diagnosis in patients with recurrent respiratory infection and atypical radiological findings such as situs inversus. Surgical management such as thoracoscopic right lower lung lobectomy requires expertise due to atypical anatomical arrangement. This case contributes to the existing literature of Kartagener's syndrome in pediatric patients and provides its effective comprehension of surgical management and emphasizes the need for timely intervention by experienced surgeons.

## Key Clinical Message

In patients with chronic sinusitis, bronchiectasis, and recurrent respiratory infection, clinicians should consider Kartagener's syndrome and evaluate for situs inversus to avoid diagnostic delays and seek management accordingly.

## 1 Introduction

Kartagener's syndrome is a rare ciliopathic autosomal recessive genetic disorder that occurs with the estimated incidence rate of 1/20,000 to 1/60,000 live births [1]. It combines the clinical triad of situs inversus, chronic sinusitis, and bronchiectasis. Kartagener's syndrome results from primary ciliary dyskinesia (PCD), leading to decreased mucosal scavenging capability and increased infection susceptibility in the respiratory tract that will cause progressive symptoms like productive cough, wheezing, and shortness of breath [2]. In its respiratory form, cilia dysfunction in the upper and lower respiratory mucosa leads to early onset of chronic obstructive pulmonary disease, secondary to mucociliary clearance disorders, leading to progressive deterioration of lung function [1]. Cardiovascular involvement is characterized by situs inversus and other congenital cardiovascular abnormalities like bilateral superior vena cava, inferior vena cava drainage site abnormality, and Tetralogy of Fallot [3].

The diagnosis of Kartagener's syndrome in early stage is very important because there is a tendency of recurrent airway tract infections in people with this syndrome, which needs to be addressed early to minimize the complications brought by infections including respiratory failure [2], it will also help to improve quality of life and life expectancy. The diagnostic parameters of this disease include clinical findings and ciliary function tests on the basis of exhaled nasal nitric oxide, bronchial biopsy, electron microscopy, and genetic studies in which bi‐allelic *DNAI1 and DNAH5* mutation tests, complete blood count (CBC), sputum analysis, prothrombin time along with chest X‐ray and chest computerized tomography (CT scan) that will show the lobar malformation of the lungs [2, 4].

The combined efforts of the patient's family and medical fraternity will help to improve the condition of the patient by maintaining lung function without developing complications. Sometimes it may demand lobectomy or lung transplantation as a permanent solution. This article has reported a rare case of Kartagener's syndrome with the triad of chronic sinusitis, situs inversus, and bronchiectasis in a 9‐year‐old female, having clinical and para clinical aspects of PCD. Alongside, the diagnostic and surgical challenges in its management including right lower lung lobectomy in tertiary care hospitals of low‐resource countries like Pakistan.

Since the literature is less reported for rare cases of Kartagener's syndrome, this rare case report presents a rare pediatric case of Kartagener's syndrome managed surgically by thoracoscopic right lower lobectomy and discusses the diagnostic and surgical challenges in a low‐resource setting, in accordance with SCARE 2023 guidelines [5].

## 2 Case History and Examination

A 9‐year‐old female presented in OPD with a complaint of fever, breathlessness, and productive cough for 3 days. Fever was undocumented, intermittent in nature, and febrile to touch. It was associated with rigors and chills. She also had complaints of breathlessness and cough. The cough was persistent and productive in nature without haemoptysis; the color of sputum was yellowish green. It was aggravating to lie down in a supine position. Past medical history showed that she had pulmonary tuberculosis when she was 1.5 years old. The systemic history of the patient was insignificant. Neither the patient nor family are smokers; and there is no history of migration. She has no asthma, sleep apnoea, or weightlessness. There is no history of parental consanguinity. There is no family history of respiratory infection or similar condition.

On general physical examination, she has mild pale palms; her fingers had definite clubbing. She has mild pale conjunctiva and no jaundice observed in her eyes. On systemic examination, all the systems were unremarkable, and respiratory examination reveals normal vesicular sounds bilaterally and coarse crackles sounds in the basal lung field.

Cardiac examination showed the apex beat in the right fifth intercostal space along the midclavicular line. Other systemic examinations were unremarkable.

## 3 Differential Diagnosis

- Cystic fibrosis (it was ruled out by using the sweat chloride test, no elevation of chlorine or pancreatic insufficiency is observed).
- Primary immunodeficiency disorders (immunoglobulin levels or serum testing were done to rule out immunodeficiency disorders.)
- Post‐tuberculous bronchiectasis

## 4 Investigations

**FIGURE 1:**
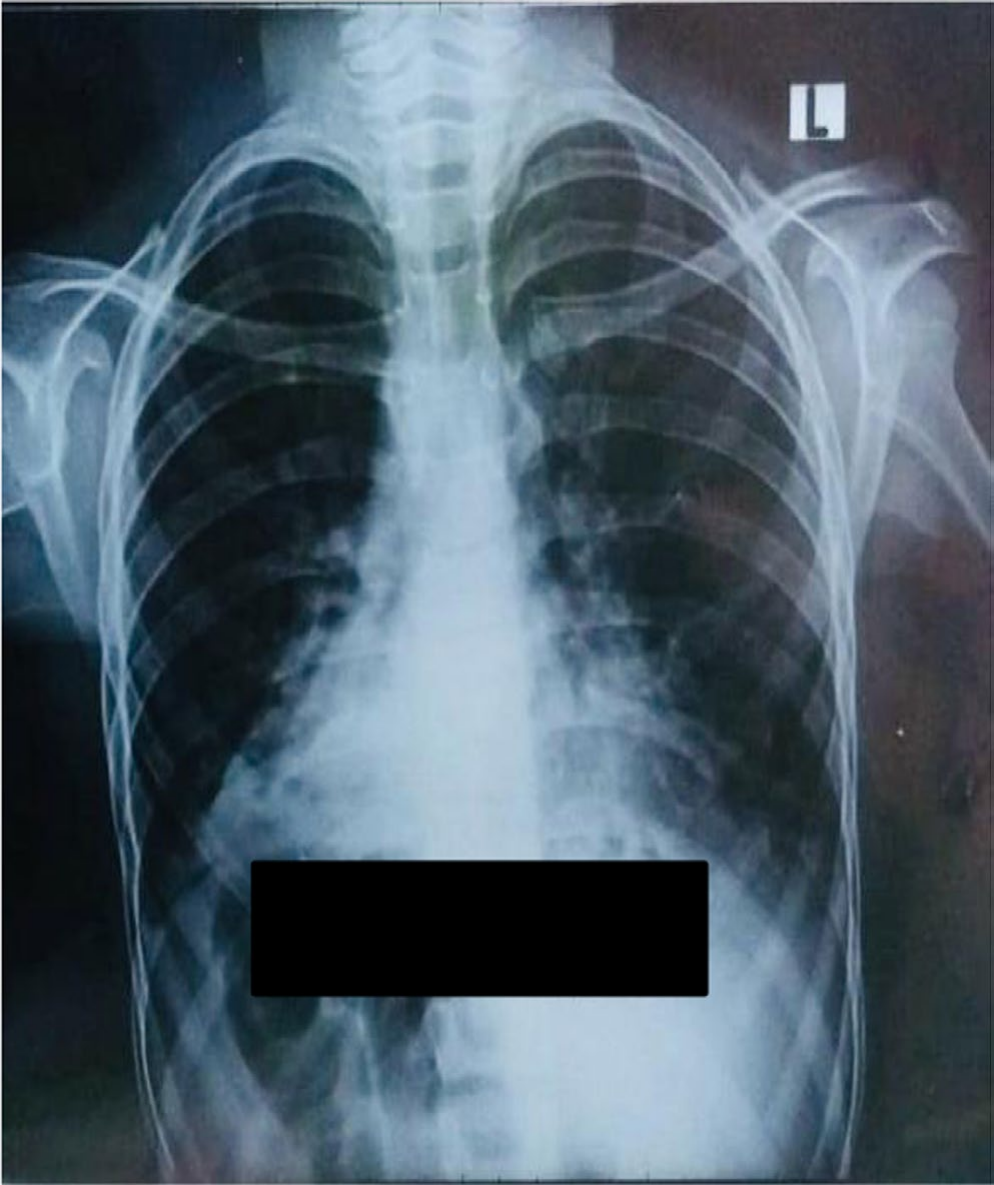
Posterio‐anterior view of chest X‐ray. This is a PA view of chest X‐ray of a 9‐year‐old girl that is showing dextrocardia and right sided stomach gas suggesting Situs inversus.

**FIGURE 2:**
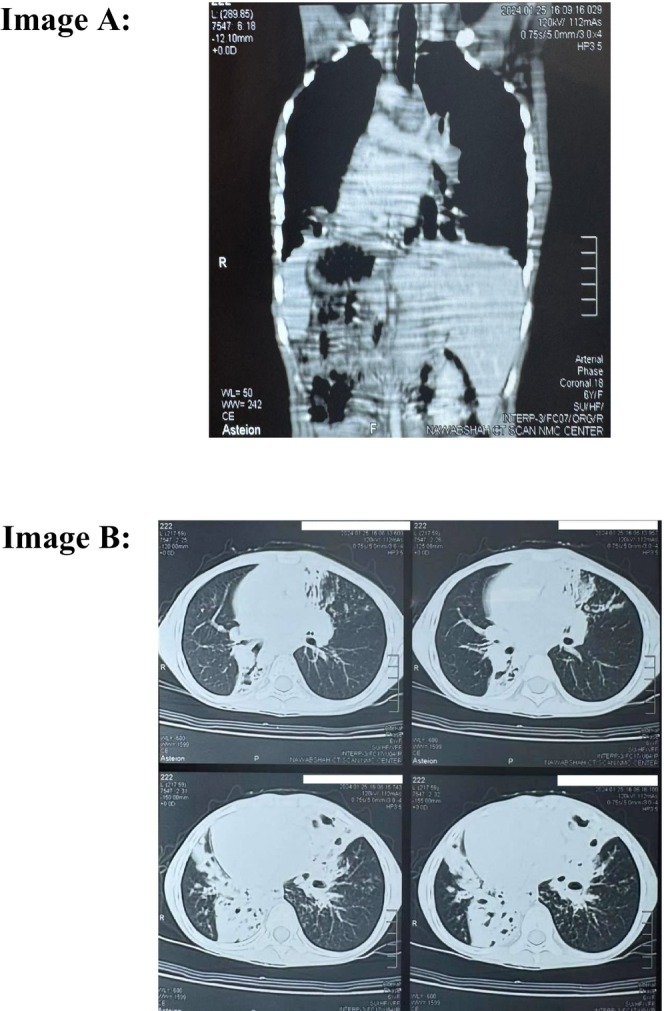
Film of chest computerized tomography. (A) Film of chest computerized tomography in coronal view which is showing Situs inversus with right side apex beat and aortic arch on right side and right lower lobe bronchiectasis. (B) Film of chest computerized tomography in axial view.

**FIGURE 3:**
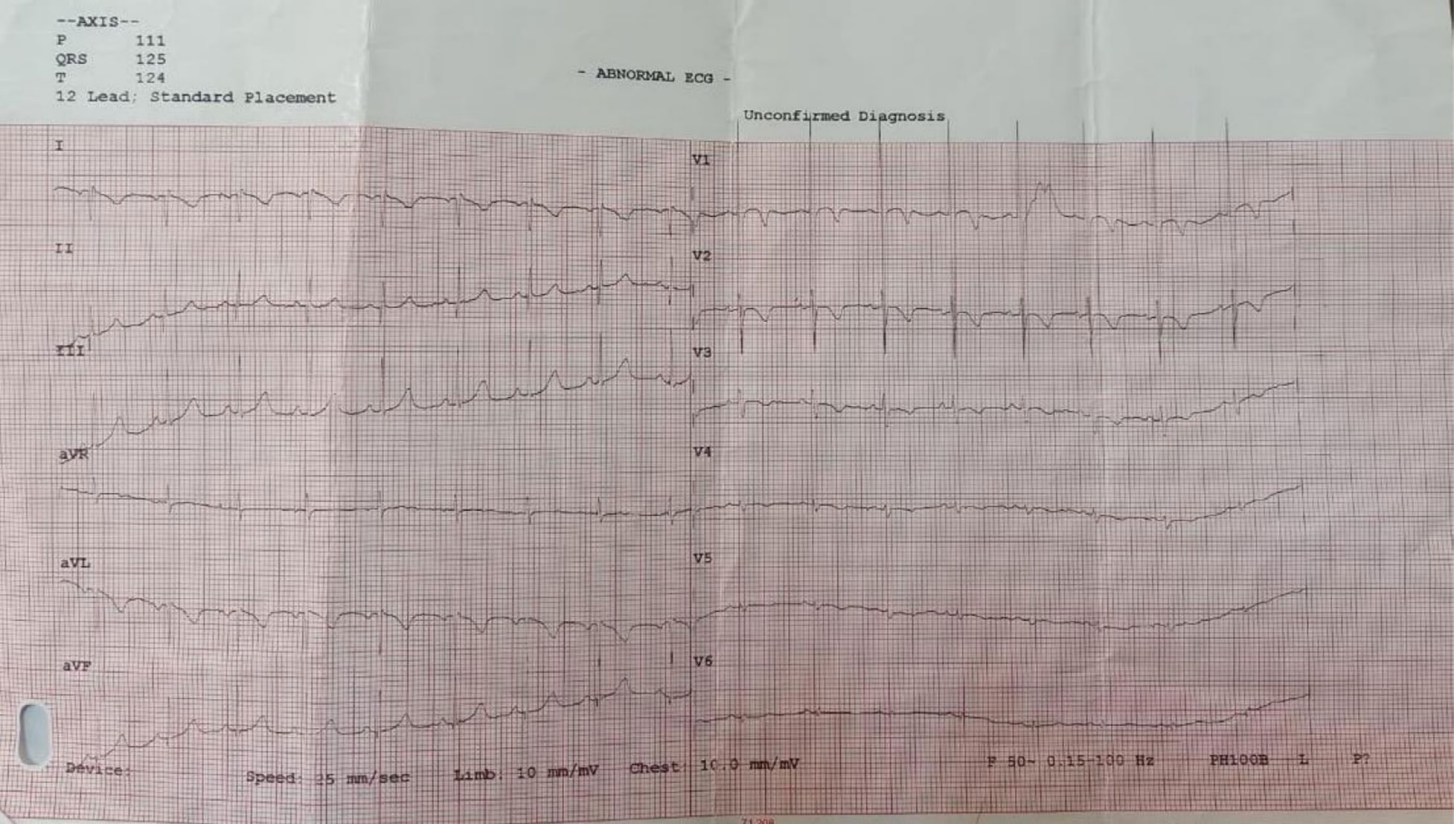
Electrocardiography of patient. The Electrocardiography of the patient revealed globally negative Lead I and aVL and globally positive aVR.

In laboratory investigations, the CBC shows mild low hemoglobin and leukocytopenia [Hb: 10.4 g/dL, TLC: 4.2 × 10^9^]. Serum electrolytes, renal, and liver function test were normal. Arterial blood gases show hypercapnic respiratory failure pH 7.3, PaCO_2_: 58 mmHg, PaO_2_ mHg: 56, HCO_3_: 28 mEq/L. Arterial blood gases demonstrated hypercapnic respiratory failure. Chest X‐ray and chest computerized tomography were advised which revealed dextrocardia and situs inversus with right side apex beat as observed in Figures 1 and 2, respectively. The electrocardiography of patient revealed globally negative Lead I and aVL and globally positive aVR with reverse progression of R wave in precordial leads as shown in Figure 3. The CT of paranasal sinuses reveals minimal mucosal thickening in ethmoid, sphenoid, maxillary sinuses. Montoux test for tuberculosis was negative. The final diagnosis of Kartagener's syndrome was made on basis of clinical signs, symptoms, and radiological findings.

## 5 Treatment

**FIGURE 4:**
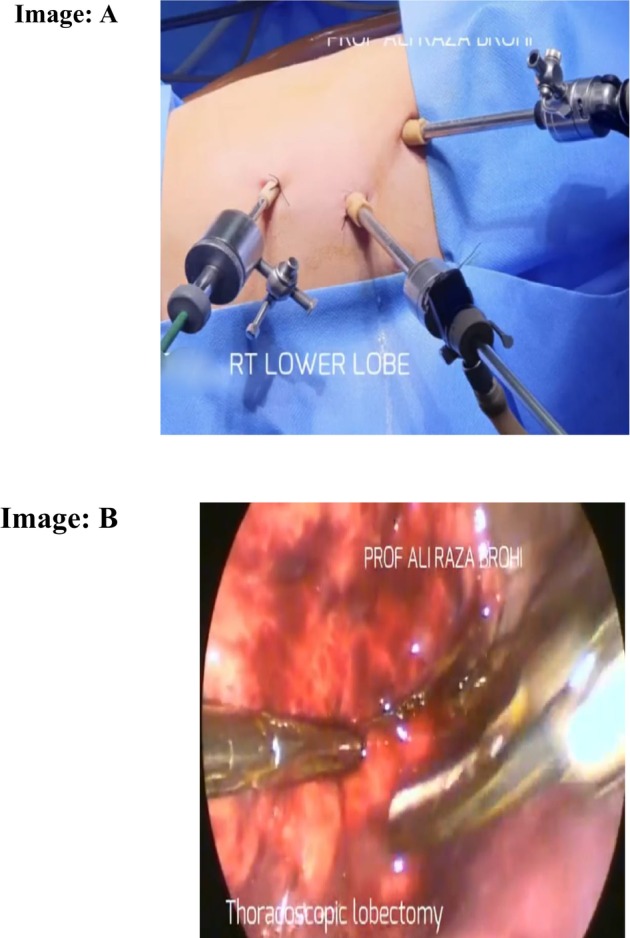
Intraoperative view of patient inside and outside of body: (A) Port Insertion on right side of chest; (B) intraoperative view of lung with bronchiectasis.

During the hospitalization course, the patient was managed conservatively with nebulization (atem 1 cc) and antibiotics including Inj: Foutem 500 mg IV, Inj: Ampicillin 500 mg IV 8H°, and 2 cc Normal saline 6H° on 1st 4 days. The antibiotic Ampicillin is given as standard treatment in respiratory infection. Conservative treatment was given to the patient for rhinosinusitis and to resolve the threatening condition of the patient. After 1 week of conservative therapy at our hospital, the patient had undergone thoracoscopic right lower lobectomy for Kartagener's Syndrome secondary to localized bronchiectasis as observed in Figure 4. The intraoperative procedure was performed by experienced laparoscopic thoracoscopic pediatric surgeons to avoid complications arising from misleading the surgical procedure due to anatomical variation in the patient. After surgery, the patient moved to ICU for continuous mechanical ventilation (low PEEP, high flow). After 3 days, the patient's symptoms improved and was discharged with prescription of oral therapy including Ciprofloxacin 15 mg/kg, every 12 h for 2 weeks, physiotherapy for 2 weeks, and follow‐up every 15 days for 6 months. On a 2 month follow‐up, the patient was alright with no complications. Moreover, this is a single case study, not to be generalized for every Kartagener's syndrome case.

## 6 Conclusion and Results (Outcome and Follow‐Up)

This case report enriches the literature on Kartagener's syndrome, a rare disorder characterized by recurrent respiratory infections and situs inversus, specifically in the pediatric age group. It is enlightening the significance of timely diagnosis and effective surgical management by experienced surgeons. Our findings are emphasizing consideration of Kartagener's syndrome in the differential diagnosis of patients with chronic respiratory symptoms and dextrocardia on chest X‐ray.

## 7 Discussion

This case report describes a case of a 9‐year‐old female diagnosed with Kartagener's syndrome on the basis of X‐ray, CT, and ECG findings, suggesting a classical triad of chronic sinusitis, situs inversus, and bronchiectasis. After receiving supportive care, the patient had undergone thoracoscopic right lower lobectomy because of her localized bronchiectasis. After surgery, during follow‐up, her symptoms were considerably improved. Same as in a case report by Lin et al., where a patient with Kartagener's Syndrome had a successful outcome after undergoing a left middle lobectomy for medically resistant bronchiectasis. These findings imply that in certain patients with localized disease that is not responding to medicinal therapy, lobectomies may be a reasonable course of treatment [6].

KS is a rare, autosomal recessive ciliopathy. It is inherited form is termed Primary Ciliary Dyskinesia (PCD), and approximately 50% of individuals with PCD exhibit situs inversus where all visceral structures are mirrored, or situs solitus, where only dextrocardia is present [7]. Cilia are essential for the coordinated movement of mucus, sperm motility, and visceral orientation during embryogenesis [8]. Mutations in genes such as DNAI1 and DNAH5, which encode components of axonemal dynein arms, may also result in dysfunctional ciliary motility. Defects may involve the radial spoke, central microtubules, inner dynein arm, or outer dynein arm [9].

Kartagener's syndrome typically manifests in childhood with chronic respiratory symptoms [10]. The diagnosis of KS is based on a combination of clinical history and diagnostic findings including dextrocardia in the patient or a sibling, immotile but viable spermatozoa, impaired tracheobronchial clearance, or ultrastructural ciliary abnormalities on electron microscopy. Additionally, the findings on screening tests include markedly reduced nasal nitric oxide, prolonged saccharin clearance time, and reduced ciliary beat frequency. There could be absent or defective dynein arms on TEM and mutations in DNAI1 and DNAH5 on genetic testing. However, there is currently no universally accepted gold standard that offers sensitivity and specificity [11]. Similar to our finding, situs inversus is a hallmark feature of KS, confirmed radiologically, along with mucosal thickening and obstruction of the osteomeatal complex, which are common CT findings in chronic sinusitis, which were minimally present in our patient [11, 12]. Additionally, there is chronic cough and a recurrent history of infection as reported in pediatric cases by Tadesse et al. [11].

The management of Kartagener's syndrome is mainly symptom based and preventive, often requiring a multidisciplinary approach. Treatment focuses on airway clearance (e.g., bronchial drainage physiotherapy), nasal irrigation, and antibiotic therapy for infections [13]. Although surgical intervention is not routinely indicated in KS, however, surgical resection was deemed appropriate for localized bronchiectasis to prevent further pulmonary complications and improve the patient's quality of life [6]. In our context, the patient underwent thoracoscopic right lower lobectomy due to localized bronchiectasis and showed marked postoperative improvement during the follow‐up period.

The limitation to our case report includes lack of confirming techniques such as ciliary ultrastructure analysis or genetic mutation testing due to low resource setting. Additionally, the evaluation of long‐term follow‐up is necessary for better investigation of patient outcomes. Since our study has reported one case, further longitudinal studies are required to understand the diagnostic process and management.

## Author Contributions

**Pakeezah Tabasum:** conceptualization, writing – original draft. **Makhdooom Bilawal:** writing – original draft, writing – review and editing. **Priya Devi:** writing – review and editing. **Waseem Sajjad:** resources, writing – original draft. **Ali Raza Brohi:** supervision. **Mohammed Hammad Jaber Amin:** resources, writing – original draft.

## Funding

The authors have nothing to report.

## Consent

The authors certify that an informed written consent was taken from the patient's parents to use his data and images for the sake of research and publication. It was informed to the patient and his parents that his name and identity will be kept anonymous while publishing the report. Consent to Publish Case Report: All authors confirm that we have obtained written informed consent from the patient's parents for the publication of this case report. As the corresponding author, I hereby grant permission to publish this case report in your Journal.

## Conflicts of Interest

The authors declare no conflicts of interest.

## Data Availability

The authors confirm that the data supporting the findings of this study are available within the article. Raw data that support the findings of this study are available from the corresponding author, upon reasonable request.
